# High-elevational occurrence of two tick species, *Ixodes ricinus* and *I. trianguliceps*, at their northern distribution range

**DOI:** 10.1186/s13071-021-04604-w

**Published:** 2021-03-18

**Authors:** Nicolas De Pelsmaeker, Lars Korslund, Øyvind Steifetten

**Affiliations:** 1 Department of Nature, Health and the Environment, University of South-Eastern Norway, Bø, Norway; 2 Department of Natural Sciences, University of Agder, Kristiansand, Norway

**Keywords:** Ticks, Altitude, *Ixodes ricinus*, *Ixodes trianguliceps*, Bank vole, *Myodes glareolus*, Distribution, Range shift

## Abstract

**Background:**

During the last decades a northward and upward range shift has been observed among many organisms across different taxa. In the northern hemisphere, ticks have been observed to have increased their latitudinal and altitudinal range limit. However, the elevational expansion at its northern distribution range remains largely unstudied. In this study we investigated the altitudinal distribution of the exophilic *Ixodes ricinus* and endophilic *I. trianguliceps* on two mountain slopes in Norway by assessing larval infestation rates on bank voles (*Myodes glareolus*).

**Methods:**

During 2017 and 2018, 1325 bank voles were captured during the spring, summer and autumn at ten trapping stations ranging from 100 m to 1000 m.a.s.l. in two study areas in southern Norway. We used generalized logistic regression models to estimate the prevalence of infestation of both tick species along gradients of altitude, considering study area, collection year and season, temperature, humidity and altitude interactions as extrinsic variables, and host body mass and sex as intrinsic predictor variables.

**Results:**

We found that both *I. ricinus* and *I. trianguliceps* infested bank voles at altitudes up to 1000 m.a.s.l., which is a substantial increase in altitude compared to previous findings for *I. ricinus* in this region. The infestation rates declined more rapidly with increasing altitude for *I. ricinus* compared to *I. trianguliceps*, indicating that the endophilic ecology of *I. trianguliceps* may provide shelter from limiting factors tied to altitude. Seasonal effects limited the occurrence of *I. ricinus* during autumn, but *I. trianguliceps* was found to infest rodents at all altitudes during all seasons of both years.

**Conclusions:**

This study provides new insights into the altitudinal distribution of two tick species at their northern distribution range, one with the potential to transmit zoonotic pathogens to both humans and livestock. With warming temperatures predicted to increase, and especially so in the northern regions, the risk of tick-borne infections is likely to become a concern at increasingly higher altitudes in the future.
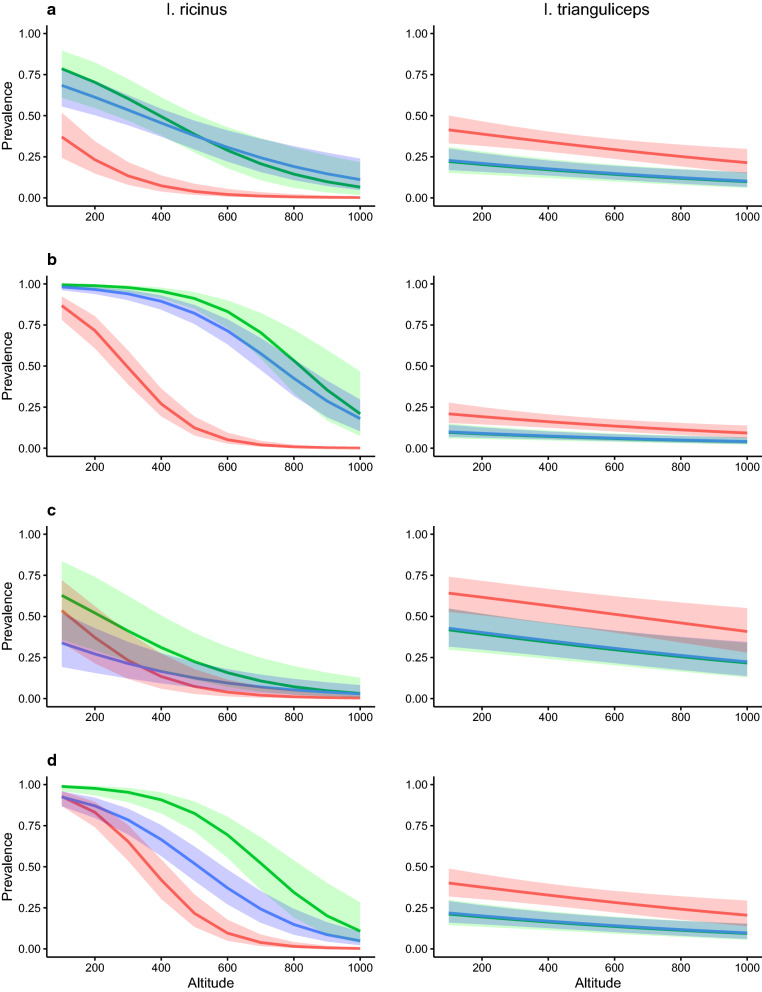

## Background

During the last decades increasing temperatures have been shown to have an impact on the distribution of species across a wide range of taxonomic groups [1, 2]. Depending on the species’ adaptability to a warmer climate and the ability to disperse, species may either (i) increase their distribution range due to conditions becoming more favourable in areas that were previously inhospitable [3, 4]; (ii) contract in range as their habitat becomes increasingly unsuitable [5, 6]; or (iii) move away from areas that have become unsuitable towards habitats that have become more favourable [7, 8]. To date most species appear to expand their natural distribution range, and for the majority of species both a northward [9, 10] and an upward [11–13] range expansion seems to be the most common movement pattern.

Along with other organisms, certain ectoparasites, such as ticks, have undergone similar range expansions [14–16]. Ticks are generally limited in their distribution by environmental factors, such as temperature and humidity [17, 18], but as hematophagous parasites they also depend on the presence of adequate hosts [19]. With a changing climate, ticks have been observed to have increased their distribution range northwards in the northern hemisphere [20–22], and several studies have also demonstrated the occurrence of ticks at increasingly higher altitudes in central Europe [14, 23, 24]. For example, in Switzerland, *Ixodes ricinus* has been recorded at altitudes up to 1070 m.a.s.l. [25, 26], and in the Czech Republic this species has shifted its altitudinal range limit up to 700 m.a.s.l. since the 1990s [27], and in one instance up to 1100 m.a.s.l. [28]. According to Danielova et al. [29], it may survive up to 1200 m.a.s.l. or higher if the habitat is favourable. Another tick species, *I. trianguliceps,* has been recorded at altitudes as high as 2300 m.a.s.l. in Switzerland [30], but because it does not quest in open vegetation like *I. ricinus*, it is likely to be less exposed to limiting environmental conditions, especially temperature, and hence able to survive in more extreme environments [19]. With any upward shift in range limit, the risk of exposure to tick-borne infections is likely to increase for both humans and livestock [24, 28], and is predicted to further increase in the future [31–33].

Most studies investigating the altitudinal dynamics of *I. ricinus* have occurred at its geographical center in Europe [14, 26, 29, 34], and very little is known about its altitudinal distribution at the northern range. Because the effects of climate change are expected to be stronger at higher latitudes [35], the increase in altitude expansion is likely to be more pronounced in Scandinavia. In Norway, *I. ricinus* has undergone a northward range shift similar to that observed in other Scandinavian countries [36–38], with the shift found as far north as 69°N [15] and considered to be permanently established at 66°N [39]. It has also expanded upwards in altitude and based on direct and indirect multi-source analysis reported by citizens, hunters, health professionals and veterinarians, has been observed up to 583 m.a.s.l. [15]. Even so, data are scarce, and the occurrence of ticks in relation to altitude and its range boundary remain largely unknown.

To the best of our knowledge, no recent field studies have investigated the altitudinal distribution of ticks at their northern distribution range in Europe. The aims of this study are to determine the altitudinal distribution patterns of the generalist tick *I. ricinus* and the specialist tick *I. trianguliceps* in Norway, by studying the infestation rates of both species on a commonly found rodent, the bank vole (*Myodes glareolus*). Because of the nest-dwelling behaviour of *I. trianguliceps*, we expect it to be less limited by altitude than *I. ricinus*.

## Methods

### Study areas

**Fig. 1:**
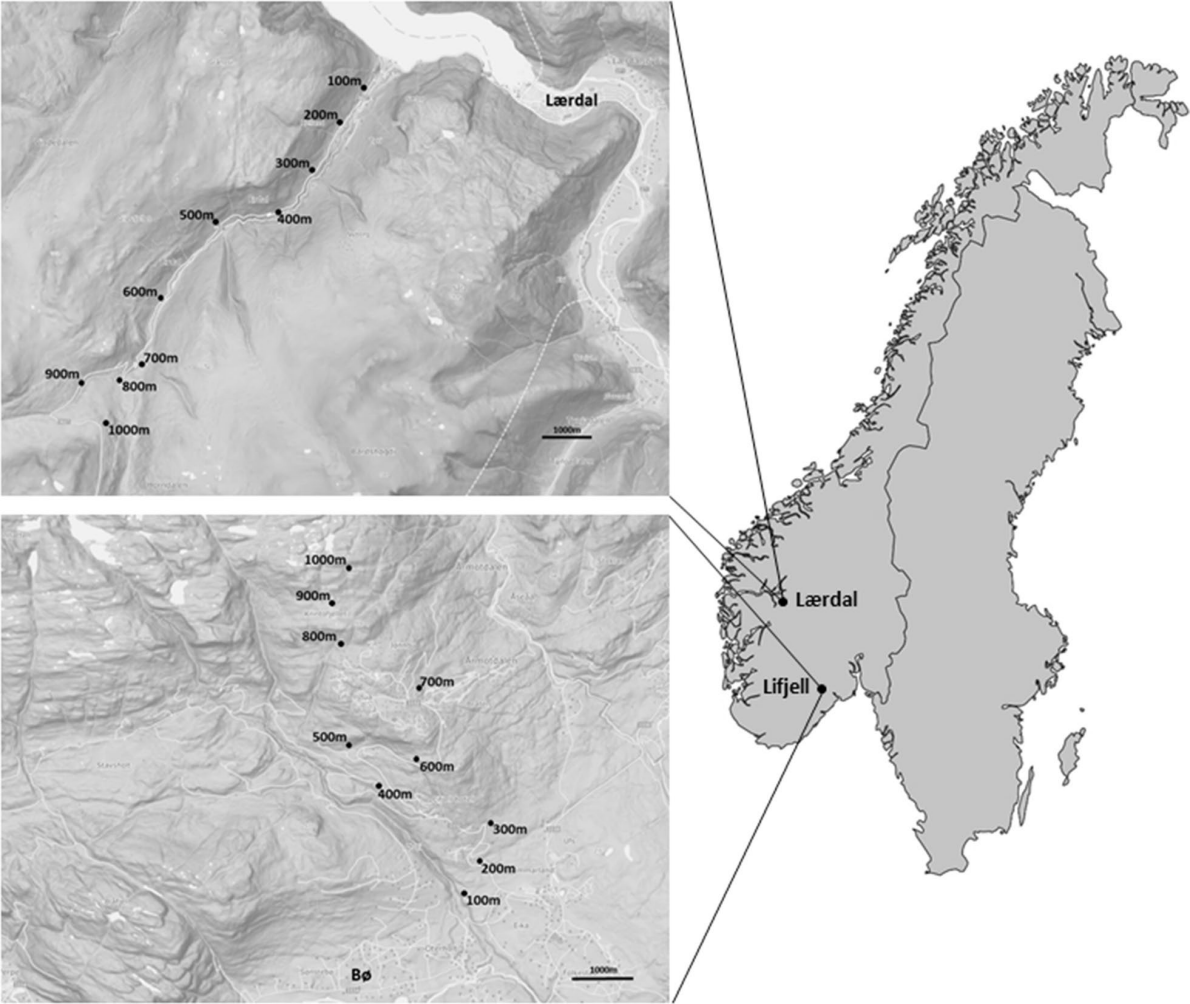
Location of the two study areas in Norway. Inset maps show the exact location of each trapping station along the altitudinal gradient in Lifjell and Lærdal. Each trapping station was placed at every 100-m altitude interval, ranging from 100 to 1000 m.a.s.l.

This study was carried out along two mountain slopes in Norway during 2017 and 2018 (Fig. 1). The first study area was a south-facing mountain slope on the Lifjell massif (59°26.495′N, 9°0.603′E), north of Bø i Telemark. It is characterized by a continental climate, located within the boreonemoral to southern boreal zone. Below the tree line, which is situated between 800 and 900 m.a.s.l., the vegetation is a blend of deciduous and coniferous forests with birch (*Betula pubescens*) and spruce (*Picea abies*) as the dominant tree species, and blueberries (*Vaccinium myrtillus*) as the dominant species at ground layer. Below the tree line the vegetation is mostly homogeneous. Above the tree line the vegetation is primarily dominated by common heather (*Calluna vulgaris*) and blueberries. Boulder fields occur frequently throughout the gradient, and the highest peak found on the plateau is 1288 m.a.s.l. Most of the data collection points were located on topographically open hillside. Temperature and precipitation normals for the study areas can be found in Appendix 1. The second study area was located in the Erdal valley (61°05.817′N, 7°24.688′E) near Lærdalsøyri (hereafter referred to as Lærdal). It is a north-facing mountain slope close to the innermost part of the Sognefjorden fjord, approximately 150 km east of the western coastline. Due to its proximity to the fjord, the climate is more maritime than that at Lifjell, characterized by cooler summers and milder winters (Appendix 1). Sampling points were mostly located within the valley formed by the Erdal river. The tree line here is situated between 900 and 1000 ma.s.l., and below the tree line the vegetation consists primarily of homogeneous deciduous forests with birch and alder (*Alnus glutinosa*) as the dominant tree species. At ground layer the vegetation is dominated by blueberries, and different species of ferns and tall perennial herbs. Above the tree line common heather, dwarf birch (*Betula nana*), common juniper (*Juniperus communis*) and crowberry (*Empetrum nigrum*) are the dominant species. Surrounding the study area are several mountain peaks exceeding 1500 m.a.s.l.

### Study species

*Ixodes ricinus* is the most common and the most studied tick species in Europe [18]. It ranges latitudinally from North Africa to Scandinavia, and longitudinally from Ireland to Russia [40]. It is a three-host tick that feeds on a wide range of mammals, birds and reptiles [41]. Immature life stages (larvae and nymphs) parasitize small mammals in larger proportions [42], whereas adults tend to feed on larger mammals [41]. It is common to deciduous and, to some extent, coniferous forests and is dependent on sufficient temperature and humidity to be able to quest (actively seeking a host). It is sensitive to desiccation and temperature extremes [43].

*Ixodes trianguliceps* occurs throughout Europe, ranging latitudinally from Italy up to well above the Arctic Circle [44]. Contrary to *I. ricinus*, *I. trianguliceps* specializes primarily on rodents and other small mammals during all life stages [45, 46]. It is endophilic (nest-dwelling), spending its off-host time within the burrows of its host to moult and quest [47]. It occurs in widely different habitats, ranging from meadows, peat bogs to dark-coniferous forests, mixed and deciduous forests, as well as high-altitude treeless zones [42, 45]. It is generally considered to be one of the most cold-resistant ticks of the genus *Ixodes* in the Palearctic region [45]. Since it rarely infests humans or livestock due to its host-seeking behaviour [48]*,* cases of tick-borne infection are considered exceptional [15], but it does contribute to maintaining the infection cycle of several pathogens between *I. ricinus* and their hosts, such as *Borrelia burgdorferi* [49] and *Anaplasma phagocytophilum* [50].

The bank vole is a commonly found rodent throughout Europe and occurs virtually everywhere in Fennoscandia [51]. It is the most common rodent species in both study areas. Other small mammal species present in the study areas are the field vole (*Microtus agrestis*), tundra vole (*M. oeconomus*), grey red-backed vole (*Myodes rufocanus*), wood mouse (*Apodemus sylvaticus*), yellow-necked mouse (*A. flavicollis*) and house mouse (*Mus musculus*). The common shrew (*Sorex araneus*), pigmy shrew (*S. minutus*) and water shrew (*Neomys fodiens*) are also found in these areas. The bank vole is a reservoir host for several tick-borne pathogens, such as *B. burgdorferi* [52], *Babesia microti* [53], *Candidatus* Neoehrlichia mikurensis [54] and *A. phagocytophylum* [55]. It is possibly the most important host for all life stages of *I. trianguliceps* [42] and is heavily infested by the immature stages of *I. ricinus* [56, 57]. Because it was common at both study areas and at all altitudes, we chose to base our analysis on this species.

### Host trapping

Ticks are commonly collected by two methods: cloth-dragging for questing (unfed) ticks and host examination for feeding ticks. The cloth-dragging method is only applicable for the collection of exophilic species, and only when the vegetation is dry. During most days of data collection the vegetation was either partly wet or weather conditions did not allow for cloth-dragging. Additionally, as we aimed to include the infestations of *I. trianguliceps* in this study, we focused solely on the capturing of hosts. However, examining tick burdens accurately from live small mammals can be difficult [58]; therefore, we opted for a combination of lethal traps and euthanized live captures. In both study areas, ten trapping stations were set up along a vertical gradient ranging from 100 to 1000 m.a.s.l. at every 100-m altitude interval. Bank voles have relatively small home ranges [59], and altitude trapping stations were located several hundred meters from each other; hence we can be reasonably confident that ticks collected from a host at a certain altitude were also acquired in the immediate vicinity. At each trapping station, two plots of 20 traps each were deployed, one plot with live traps (Ugglan Special Nr. 2; Grahnab AB, Gnosjö, Sweden; http://www.grahnab.se), and the other plot with lethal traps (Rapp2 Mousetrap; Nordenfjeldske Børstefabrikk AS, Surnadal, Norway; http://www.rappfellene.no). The traps in both plots were arranged in a 4 × 5 grid, with a spacing of 10 m between each trap. Live traps were baited with a slice of apple for hydration and whole oats for caloric value, and a lining of sawdust was provided on the trap floor as insulation. Lethal traps were baited with peanut butter for practical reasons as it is easily applied to the inside of the trap body. Trap type does not influence tick burden size on the captured animals [60]. At each altitude, the live and lethal plots were placed at approximately 100 m distance from each other, but in locations with similar vegetation structure and habitat characteristics. As humidity and temperature have a direct influence on tick activity, they are important drivers of phenological patterns and host-seeking behaviour [61]. For this reason, a datalogger (TinyTag Plus 2–TGP 4017, housed in a DataMate ACS-5050 instrument cover; Hastings Data Loggers, Port Macquarie, Australia) was placed in between the two plots at each trapping station, approximately 50 cm above ground level, for measuring air temperature and relative air humidity at 1-h intervals for the duration of the trapping period. The height of 50 cm was chosen to capture the general variation in environmental conditions at each altitude. Trapping took place during the spring (20–30 May), summer (20-30 July) and autumn (20–30 September) seasons of 2017 and 2018. As an exception, during the spring season of 2017, capturing took place from 1 to 7 June, and only up to 700 m.a.s.l. in both areas, as there was too much snow to allow for the operation of traps earlier and above this altitude. During each trapping period, traps were checked every 24 h, and the collection of trapped animals started at 08:30 h. When checking the trapping grids, triggered lethal traps were rebaited and reset. As examining live small mammals for ticks can be stressful and cause injury or death [62], all animals captured in live traps were euthanized by cervical dislocation of the head upon collection, and each individual was kept separately in a sealed and coded plastic bags. Activated live traps were emptied of the remaining contents, and new insulation and food were provided before resetting the traps. At the end of every collection day, all animals were placed in a freezer at − 20 °C.

### Laboratory processing

At the end of every trapping season, captured bank voles were examined for ticks in the laboratory, as a full-body post-mortem examination provides the highest degree of sensitivity [63]. The day before the examination, the voles were removed from the freezer and left to thaw overnight at 10 °C. The voles were examined one by one and taken out of the plastic bags individually. The empty bags were checked for ticks that might have dropped off. It was our observation that a number of ticks would drop off the host when the animals were placed in the freezer, possibly in an attempt to escape the extreme temperatures. Animals that were wet were dried with a hairdryer before examination. The hosts were checked for ticks starting with the head, ears and snout, followed by the neck and throat, back and abdomen, legs, feet and tail. Attached or detached ticks were removed from the host using tweezers. Collected ticks were removed and placed in a 1.5-ml plastic Eppendorf tube containing a 70 % ethanol solution (1 vial per host). Finally, a lice comb was brushed through the fur of the animal from tail to head (against the hair orientation), and the vole was shaken by the tail above a white plastic tray to collect any ticks that might have been missed during the examination. The hosts were then weighed to the nearest tenth of a gram, and the sex was determined. The minimum amount of time needed to process one animal was 20 min. After the examination, the animals were bagged in new plastic bags and refrozen at − 20 °C.

Ticks were determined for life stage and species under a Zeiss Discovery V20 stereomicroscope (Carl Zeiss AG, Oberkochen, Germany), using an established publication key as reference [64]. Because more than 94 and 75% of all *I. ricinus* and *I. trianguliceps* collected, respectively, were larvae, only the larval stage was included in the analysis.

### Data analysis

The statistical analyses were performed using the software package R version 3.5.3 [65]. The analysis of *I. ricinus* larvae and *I. trianguliceps* larvae was performed separately. As is usually the case with tick presence on small mammals, neither tick species was evenly distributed on the hosts [66], and 13.8 and 82.0 % of the hosts had no *I. ricinus* and *I. trianguliceps* larvae present, respectively. We therefore chose to use the presence or absence of larvae as the response variable and applied generalized linear modelling with a binomial distribution, i.e. logistic regression. The probability of encountering a tick was defined as prevalence. As predictor variables we considered altitude (ranging from 100 to 1000 m.a.s.l., as a continuous variable), study area (Lifjell and Lærdal), collection year (2017 and 2018), season (spring, summer and autumn) and humidity (%). Because altitude and temperature were negatively correlated (Pearson correlation test:* t* = − 8.1578, *df* = 1323,* p* < 0.001,* r* = − 0.219), temperature per se was not used as a predictor variable in order to avoid introducing collinearity into the model. Two-way interactions between season and altitude, study area and altitude, year and altitude, study area and year, as well as year and season were included in the starting model. We also considered individual bank vole body mass (in grams) and sex (male or female) as intrinsic co-variates. Starting with a full starting model containing all variables and the two-way interactions listed above, we used a backward step model selection process to progressively remove non-significant predictors, by comparing the residual deviance and degrees of freedom of nested models using a Chi-square test (*χ*^2^), until an optimal model that contained only significant predictor variables (*α* = 0.05) was found. To visually represent the infestation probabilities of both species across study areas, collection years and seasons, we created multiple regression line plots using the *ggplot2* package in R [67].

### Ethics statement

This study was carried out in strict accordance with regulations issued by the Norwegian Environment Agency, and a permit was provided prior to the start of the sampling (Miljødirektoratet, reference number: 2017/4651) for the duration of the trapping period. The trapping protocol for animal capture was approved by the Animal Ethics Committee of the Department of Nature, Health and Environment (University of South-Eastern Norway). All efforts were made to minimize animal suffering.

## Results

**Fig. 2:**
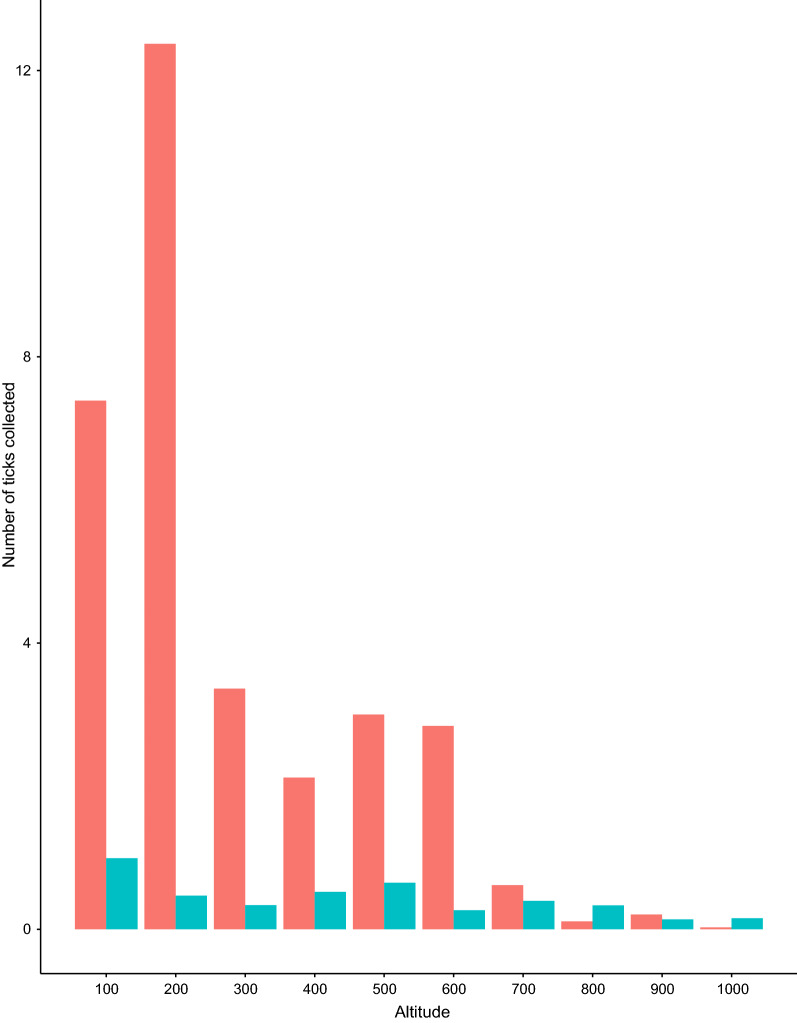
Total number of tick larvae collected from bank voles (*Myodes glareolus*) per altitude (m a.s.l.), for both study areas, years and seasons combined. Red bars: *Ixodes ricinus*; blue bars: *I. trianguliceps*

During 2017 and 2018, a total of 43,920 trap nights was performed, capturing 1325 bank voles (976 and 349 captures in 2017 and 2018, respectively). Altogether 5372 tick larvae were collected from the trapped voles at all altitudes of the gradient. Larvae of *I. ricinus* were 7.4-fold more numerous than those of *I. trianguliceps* (88.1 and 11.9 % of ticks, respectively). Larval burdens ranged from one to 100 ticks for *I. ricinus* (mean ± 95% confidence interval [CI]: 3.6 ± 0.5) and from one to 29 for *I. trianguliceps* (mean ± 95% CI: 0.5 ± 0.1), and more than half of the bank voles (57.4 %) were infested with at least one larva of either tick species. For *I. ricinus* and *I. trianguliceps*, respectively, 46.8 and 18.0% of voles carried at least one larva. Of all the voles, 7.3 % were infested with at least one larva of both tick species. The total number of ticks collected from voles at every altitude is shown in Fig. 2. Mean burdens can be found in Appendix 2. An overview of all captured host species and infestation rates is listed in Appendix 3.

### *Ixodes ricinus*

**Fig. 3:**
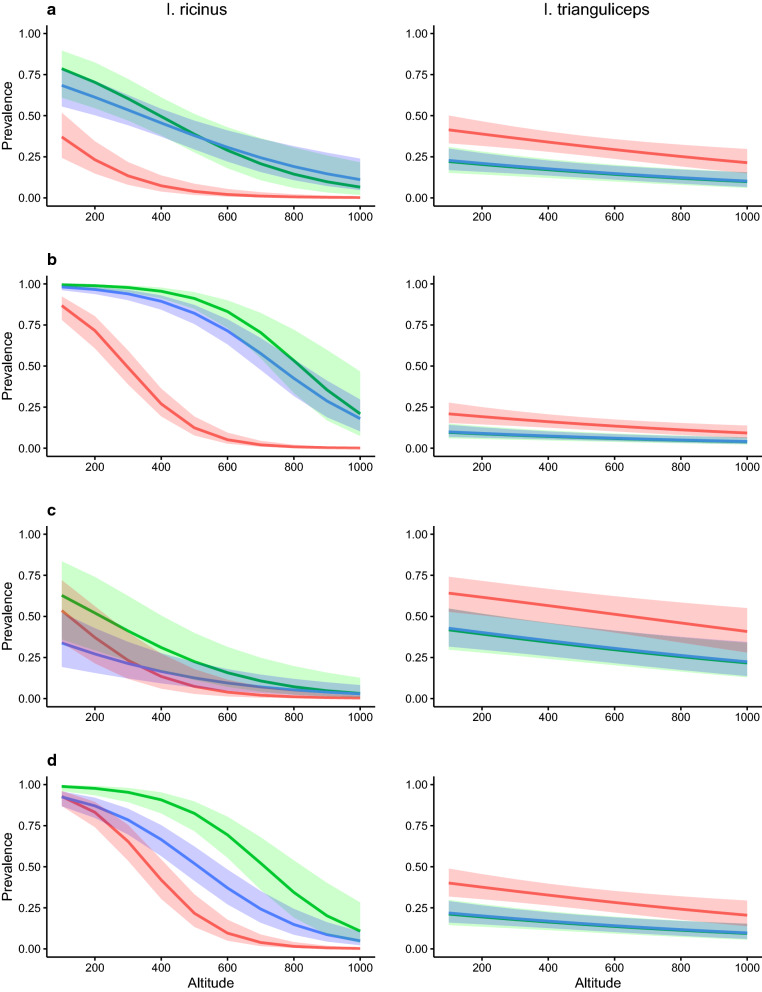
Prevalence of ticks on bank voles along an altitudinal gradient in Lifjell 2017 (**a**), Lærdal 2017 (**b**), Lifjell 2018 (**c**) and Lærdal 2018 (**d**). A prevalence of 1.0 represents a certainty of encountering at least one tick. Solid lines denote seasons: spring (green), summer (blue), autumn (red). Shaded areas around each coloured line represent the standard error 95 % confidence intervals. For *I. trianguliceps* the spring and summer curves are closely overlapping

The best model shows that there was a clear negative overall effect of altitude on the prevalence of *I. ricinus* presence (*Z* = − 3.954, *P* < 0.001), and a significant interaction between altitude and season (*χ*^2^ = 12.3, *df* = 2, *P* = 0.002) illustrates that this effect was stronger in the autumn than in the spring and summer (Table 1; Fig. 3). In addition, a significant interaction between altitude and site (*χ*^2^ = 11.1, *df* = 1, *P* < 0.001) shows that the negative altitude effect was stronger in Lærdal than in Lifjell (Table 1), driven mainly by the fact that, while the prevalence is low at 1000 m.a.s.l. at both sites, the prevalence at the lower altitudes was higher in Lærdal (close to 1) than in Lifjell (around ≤ 0.75) (Fig. 3). The model further shows that the overall prevalence of *I. ricinus* was highest among voles captured in the spring and lower, but not significantly lower, among voles captured in the summer (*Z* = − 1.14, *P* = 0.25) and significantly lower among voles captured in the autumn (*Z* = − 2.65, *P* = 0.08) (Table 1; Fig. 3). The prevalence of *I. ricinus* was overall lower in 2018 (*Z* = − 2.06, *P* = 0.04), but the seasonal effect, with lower prevalence in the autumn, was much stronger in 2017 than in 2018, as indicated by a significant interaction between year and season (*χ*^2^ = 26.7, *df* = 2, *P*  < 0.001) (Table 1; Fig. 3). Finally, the significant interaction between site and season shows that the effect of season was stronger in Lærdal than in Lifjell (*χ*^2^ = 12.3, *df* = 2, *P* = 0.002), with especially the autumn prevalence being lower in Lærdal (*Z* = − 2.73, *P* = 0.006) (Table 1; Fig. 3).

**Table 1:** Parameter estimates for the infestation prevalence on bank voles indicating the probability to be infested with at least one tick, using a binomial distribution model

Parameters	Estimate	Standard error	*Z* value	*P* value
*Ixodes ricinus* larvae				
Intercept	1.7429	0.5243	3.32	0.001
Study area Lærdal	*4.2441*	*0.5824*	*7.29*	*< 0.001*
Altitude	*− 0.0044*	*0.0011*	*− 3.95*	*< 0.001*
Sex female	*− 0.6724*	*0.1719*	*− 3.91*	*< 0.001*
Year 2018	*− 0.7789*	*0.3780*	*− 2.06*	*0.039*
Season summer	− 0.6515	0.5701	− 1.14	0.253
Season autumn	*− 1.6039*	*0.6048*	*− 2.65*	*0.008*
Altitude: season summer	0.0012	0.0012	1.03	0.305
Altitude: season autumn	− 0.0023	0.0014	− 1.59	0.112
Study area Lærdal: altitude	*− 0.0029*	*0.0009*	*− 3.39*	*0.001*
Study area Lærdal: season summer	− 0.7681	0.4656	− 1.65	0.099
Study area Lærdal: season autumn	*− 1.5402*	*0.5646*	*− 2.73*	*0.006*
Year 2018: season summer	− 0.6665	0.4740	− 1.41	0.160
Year 2018: season autumn	*1.4512*	*0.4858*	*2.99*	*0.003*
*Ixodes trianguliceps* larvae				
Intercept	− 1.1554	0.2461	− 4.70	< 0.001
Study area Lærdal	*− 0.9831*	*0.1690*	*− 5.82*	*< 0.001*
Altitude	*− 0.0011*	*0.0003*	*− 3.37*	*0.001*
Year 2018	*0.9297*	*0.1785*	*5.21*	*< 0.001*
Season summer	0.0412	0.2257	0.18	0.855
Season autumn	*0.9133*	*0.2114*	*4.32*	*< 0.001*

Values in italics indicated significance for respective parameters

Baseline for study area is Lifjell 2017, spring season and male voles

While host weight did not affect the prevalence of *I. ricinus* (*χ*^2^ = 0.106, *df* = 1, *P* = 0.75), ticks were found significantly more often found on male hosts than on female hosts, resulting in a significant sex effect in the best model (*χ*^2^ = 15.0, *df* = 1,* P* < 0.001).

### *Ixodes trianguliceps*

The best model explaining the prevalence of *I. trianguliceps* on bank voles was ultimately found to be much simpler than that for *I. ricinus* (Table 1). Also for this species there was a clear negative overall effect of altitude on prevalence (*χ*^2^ = 11.7, *df* = 1,* P* < 0.001) (Table 1); however, the overall prevalence was much lower than that found for *I. ricinus* (Fig. 3). The overall prevalence of *I. trianguliceps* was significantly lower in Lærdal than in Lifjell (*χ*^2^ = 34.7, *df* = 1, *P* < 0.001) and significantly higher in 2018 than in 2017 (*χ*^2^ = 27.0, *df* = 1, *P* <0.001) (Table 1; Fig. 3). The model further shows a clear effect of season (*χ*^2^ = 35.7,* df* = 2, *P* < 0.001), with the overall prevalence of *I. trianguliceps* being higher among voles captured in the autumn and with an equal prevalence in spring and summer (Table 1; Fig. 3).

Neither host weight (*χ*^2^ = 2.66, *df* = 1, *P* = 0.10) nor host sex (*χ*^2^ = 1.11, *df =* 1, *P* = 0.29) affected the prevalence of *I. trianguliceps* presence on bank voles.

## Discussion

The highest recorded observation for *I. ricinus* has up until now been 583 m.a.s.l. [15], but our study shows that both *I. ricinus* and *I. trianguliceps* were present at an altitude of at least 1000 m.a.s.l. in two locations located in the eastern and western part of southern Norway. We can therefore assume that in this region, both specie*s* are established at altitudes up to 1000 m.a.s.l.

### The effect of altitude on prevalence

Although both tick species decreased in abundance with increasing altitude, both were found at all altitudes of the gradient. We expected altitude to have a stronger effect on *I. ricinus* than on *I. trianguliceps*, and our results support this expectation. While the prevalence of *I. ricinus* larvae was generally higher than that of *I. trianguliceps* at low altitudes, infestation of the former showed a stronger decline with increasing altitude during all three seasons than did infestation the latter, and this seems mostly a result of a much higher prevalence of this species at lower altitudes. The strongest decline in *I. ricinus* occurred during the autumn season, when the prevalence at the top of the gradient (above 900 m.a.s.l.) was zero, while still a substantial proportion of the hosts were infested at lower altitudes. Bown et al. [68] showed that excluding roe deer (*Capreolus capreolus*) as tick hosts reduces the larval burden of *I. ricinus* on small rodents, but not that of *I. trianguliceps*. The generally higher *I. ricinus* prevalence at lower altitudes in our study might therefore be caused by *I. ricinus* being able to make use of cervids as hosts, since we expect such hosts to be more common at the lower altitudes. At higher altitudes it is possible that both tick species are limited by the lower overall temperatures and/or the shorter snow-free season, but that *I. trianguliceps* is less affected due to its endophilic nature. It is reasonable to assume that *I. ricinus* cannot be active for as many days at 1000 m as at lower altitudes, which would reduce the probability of finding a host, ultimately leading to a lower population density. *Ixodes trianguliceps*, however, seeking a small mammal host within its burrows, is not as limited by low ambient temperatures [69] and should thus be more capable of completing its life-cycle at higher altitudes. This distinction might explain the different decline with altitude for these two tick species in our study.

Persistence of tick populations at high altitudes is not necessarily restricted by survival, molting success or oviposition [17, 30]; rather, reduced hatching success is what prevents the establishment of populations [30]. The presence of feeding larvae on rodents at high altitudes in this study indicates that the two species are capable of completing their life-cycle. Because our study did not cover locations above 1000 m.a.s.l., the actual altitudinal range limit of *I. ricinus* and *I. trianguliceps* in these areas of Norway is still unknown. Nonetheless we have documented *I. ricinus* presence far above what has previously been reported [15].

### The effect of season on prevalence

For all altitudes the prevalence of *I. ricinus* was generally highest in the spring. Studies in Switzerland, Italy and the UK have shown *I. ricinus* larvae to have a bimodal activity pattern, peaking in the spring and autumn [23, 44, 70] or only peaking in early summer [71]. In our study, almost equally high prevalences were found in the spring and summer of 2017, but prevalence dropped before the summer of 2018 when conditions were somewhat drier, and although humidity was not a reliable predictor of infestation for the two species, this could explain this drop in prevalence. Overall it would appear that *I. ricinus* larvae are not considerably inhibited by summer drought in this region and that the relatively cool and humid conditions of the two regions, compared to further south in Europe, enables it to continue questing as long as temperatures are high enough.

Larvae of *I. trianguliceps* were infesting voles during all active seasons of the year, but in contrast to *I. ricinus*, prevalence was highest in the autumn. Because *I. trianguliceps* is dependent on a very humid environment [72], it is possible that generally more rain and lower temperatures in the autumn lead to more favourable conditions for *I. trianguliceps* at this part of the year.

### The effect of host characteristics on prevalence

Higher levels of parasitism of male hosts compared to female hosts is a common observation [73–75], and in this study vole sex was a determining factor in the prevalence of *I. ricinus* larvae, with males having higher infestations than females. As males roam around more than females [76, 77], the probability of males encountering ticks is higher, and this could explain the sex difference in the infestation rates of *I. ricinus*. However, no significant difference was found in infestation with *I. trianguliceps* larvae between male and female voles. As both male and female voles probably utilize burrows to a similar extent, both sexes should be equally exposed to infestations of *I. trianguliceps* larvae, which is in agreement with earlier findings [44, 46].

## Conclusions

In this study we show that both *I. ricinus* and *I. trianguliceps* are present at much higher altitudes than what has previously been documented in Norway. With ticks expanding to higher altitudes there is an increasing risk of human and livestock infection with tick-borne diseases. The occurrence of tick-borne infections has both social and economical consequences, but to date these problems have been restricted to low-altitude areas in this region. Although human cases of tick-borne infections* via* *I. trianguliceps* are rare [15], this species helps maintain pathogens in enzootic cycles of small mammals [50, 68, 78]. Several recent studies have investigated the occurrence of tick-borne pathogens in* I. ricinus* ticks in Norway [79–81], but to date no investigation has yet focussed on disease dynamics in relation to altitude. In combination with the results of this study, an in-depth survey along altitudinal gradients investigating the prevalence of pathogens in ticks could provide valuable insights into the actual disease risk to humans and livestock in Norwegian mountains. As projections indicate a continuing trend in warming temperatures, particularly in the northern regions [82], the northward and upward progression of ticks under the influence of climate change is expected to continue in the future [83]. This will further expose humans and livestock to tick-borne infections in areas where no infection risk was present before.

## Figures and Tables

**Fig. 4:**
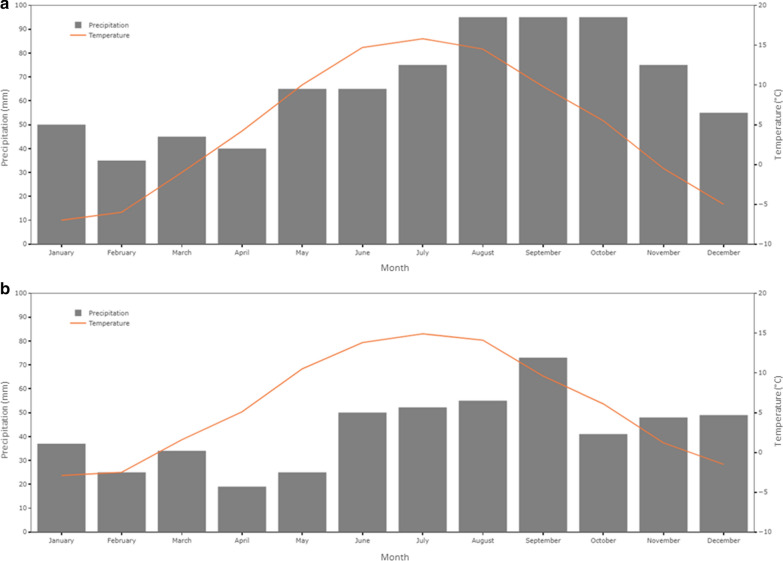
Temperature and precipitation normals in Lifjell (**a**) and Lærdal (**b**). Data were obtained from the Norwegian Meteorological Institute

## Data Availability

The dataset is deposited at usn.figshare.com. https://doi.org/10.23642/usn.12917960.

## Appendices

### Appendix 1

See Fig. 4.

### Appendix 2

See Table 2.

**Table 2:** Mean larval burden sizes of *I. ricinus* and *I. trianguliceps* found on bank voles at every altitude during 2017 and 2018 in Lifjell and Lærdal.

Altitude (m a.s.l.)	*Ixodes* sp.	Lifjell						Lærdal					
		2018			2017			2018			2017		
		Spring	Summer	Autumn	Spring	Summer	Autumn	Spring	Summer	Autumn	Spring	Summer	Autumn
100	*I. ricinus*	0.3 ± 0.7	0.8 ± 1.0	0.2 ± 0.5	19.0 ± NA	0.0 ± 0.0	4.8 ± 5.3	84.0 ± 25.2	19.7 ± 20.3	10.5 ± 9.8	43.3 ± 32.6	6.3 ± 4.6	2.1 ± 1.6
	*I. trianguliceps*	1.3 ± 2.2	0.2 ± 0.7	1.6 ± 3.4	0.0 ± NA	1.0 ± 1.4	4.6 ± 6.5	0.3 ± 0.6	0.1 ± 0.3	0.6 ± 15	0.0 ± 0.0	0.2 ± 0.4	1.6 ± 2.8
200	*I. ricinus*	15.0 ± 10.9	8.1 ± 7.4	2.9 ± 1.6	NA	NA	NA	23.8 ± 9.8	13.2 ± 9.1	8.9 ± 4.9	31.6 ± 25.0	19.4 ± 19.1	4.3 ± 3.0
	*I. trianguliceps*	0.4 ± 1.0	0.0 ± 0.0	0.9 ± 1.1	NA	NA	NA	0.0 ± 0.0	0.3 ± 0.7	0.4 ± 08	0.5 ± 0.9	0.1 ± 0.3	1.4 ± 3.5
300	*I. ricinus*	1.1 ± 0.7	1.5 ± 2.4	0.6 ± 1.4	NA	NA	NA	19.5 ± 13.7	3.1 ± 3.3	0.7 ± 1.1	10.6 ± 6.9	2.8 ± 4.0	0.5 ± 0.7
	*I. trianguliceps*	0.0 ± 0.0	0.0 ± 0.2	1.6 ± 2.8	NA	NA	NA	0.0 ± 0.0	0.0 ± 0.0	0.7 ± 14	0.2 ± 0.4	0.2 ± 0.4	0.4 ± 1.0
400	*I. ricinus*	0.9 ± 1.3	0.9 ± 1.9	0.0 ± 0.0	0.5 ± 1.0	0.0 ± 0.0	0.0 ± NA	8.1 ± 7.3	1.6 ± 1.6	0.2 ± 0.6	9.5 ± 15.3	1.0 ± 1.4	0.3 ± 0.6
	*I. trianguliceps*	0.3 ± 0.6	0.6 ± 1.1	2.3 ± 4.9	0.5 ± 0.6	0.0 ± 0.0	4.0 ± NA	0.0 ± 0.0	0.0 ± 0.2	0.3 ± 05	0.3 ± 0.5	0.6 ± 1.2	1.2 ± 3.3
500	*I. ricinus*	0.5 ± 0.8	0.1 ± 0.4	0.0 ± 0.2	0.5 ± 1.2	NA	NA	14.5 ± 8.0	4.1 ± 2.6	0.0 ± 0.2	12.7 ± 10.5	2.6 ± 3.2	1.0 ± 1.8
	*I. trianguliceps*	0.3 ± 0.6	0.5 ± 0.9	1.5 ± 3.9	1.2 ± 1.3	NA	NA	0.1 ± 0.3	0.3 ± 1.4	0.5 ± 1.9	0.1 ± 0.3	1.4 ± 4.3	1.6 ± 2.3
600	*I. ricinus*	0.2 ± 0.4	1.1 ± 1.2	0.1 ± 0.3	0.0 ± NA	0.0 ± 0.0	NA	13.8 ± 12.9	1.6 ± 1.5	0.0 ± 0.0	2.1 ± 2.6	0.4 ± 0.9	0.2 ± 0.4
	*I. trianguliceps*	0.4 ± 0.7	0.1 ± 0.9	0.5 ± 1.1	0.0 ± NA	0.5 ± 0.7	NA	0.1 ± 0.4	0.0 ± 0.0	0.1 ± 0.3	0.3 ± 0.8	0.5 ± 0.9	0.7 ± 1.1
700	*I. ricinus*	2.8 ± 7.9	0.0 ± 0.0	0.0 ± 0.0	NA	0.0 ± NA	NA	2.0 ± 3.3	1.4 ± 1.9	0.0 ± 0.0	0.3 ± 0.6	0.4 ± 0.9	0.0 ± 0.0
	*I. trianguliceps*	0.0 ± 0.0	1.3 ± 5.9	0.2 ± 0.5	NA	0.0 ± NA	NA	0.3 ± 0.8	0.1 ± 0.5	0.1 ± 0.4	0.0 ± 0.0	0.2 ± 0.4	1.1 ± 3.0
800	*I. ricinus*	NA	NA	0.0 ± 0.0	0.0 ± 0.0	NA	0.0 ± 0.0	NA	0.2 ± 0.4	0.0 ± 0.0	0.2 ± 0.4	0.4 ± 0.9	0.2 ± 0.4
	*I. trianguliceps*	NA	NA	0.2 ± 0.7	0.0 ± 0.0	NA	2.0 ± 1.4	NA	0.1 ± 0.4	0.5 ± 1.0	0.0 ± 0.0	0.0 ± 0.0	1.0 ± 2.4
900	*I. ricinus*	NA	0.1 ± 0.4	0.0 ± 0.0	NA	NA	NA	NA	0.9 ± 0.9	0.0 ± 0.0	NA	0.0 ± 0.0	NA
	*I. trianguliceps*	NA	0.1 ± 0.4	0.3 ± 0.8	NA	NA	NA	NA	0.0 ± 0.0	0.1 ± 0.6	NA	0.2 ± 0.7	NA
1000	*I. ricinus*	NA	0.2 ± 0.4	0.0 ± 0.0	NA	0.0 ± NA	0.0 ±	NA	0.0 ± 0.0	0.0 ± 0.0	NA	0.0 ± NA	0.0 ± 0.0
	*I. trianguliceps*	NA	0.0 ± 0.0	0.4 ± 0.5	NA	0.0 ± NA	0.0 ± NA	NA	0.0 ± 0.0	0.2 ± 0.4	NA	0.0 ± NA	0.0 ± 0.0

Values are presented as the mean ± standard deviation

NA indicates either that no voles were captured, or that not enough data were available to compute a standard deviation

### Appendix 3

See Table 3.

**Table 3:** Total number of hosts captured of each mammal species during 2017 and 2018, relative infestation rates and mean burden of *I. ricinus* and *I. trianguliceps*

Host species	*I. ricinus*			*I. trianguliceps*	
	No. of captures	Prevalence^a^	Mean burden (± SD)	Prevalence^a^	Mean burden (± SD)
*Apodemus flavicollis*	1	100.0	62.0 ± NA	100.0	1.0 ± NA
*Apodemus sylvaticus*	23	56.5	6.4 ± 13.5	34.8	0.9 ± 1.7
*Microtus agrestis*	130	18.5	1.7 ± 7.9	6.9	0.1 ± 0.3
*Microtus oeconomus*	26	50.0	1.5 ± 2.5	23.1	0.3 ± 0.7
*Mus musculus*	1	100.0	11.0 ± NA	0.0	0.0 ± NA
*Myodes glareolus*	1325	46.8	3.6 ± 9.0	18.0	0.5 ± 1.8
*Myodes rufocanus*	88	19.3	0.5 ± 1.4	19.3	0.6 ± 2.0
*Neomys fodiens*	2	100.0	1.0 ± 0.0	50.0	7.5 ± 10.6
*Sorex araneus*	1733	30.0	3.3 ± 12.5	20.8	0.8 ± 3.4
*Sorex minutus*	16	31.3	0.4 ± 0.7	56.3	3.0 ± 4.4

NA indicates that not enough captures were made to compute a standard deviation

^a^Prevalence is the relative infestation rate, given in percentage infestation
